# An Overview of Frog Skin-Derived Esc Peptides: Promising Multifunctional Weapons against *Pseudomonas aeruginosa*-Induced Pulmonary and Ocular Surface Infections

**DOI:** 10.3390/ijms25084400

**Published:** 2024-04-16

**Authors:** Maria Luisa Mangoni, Maria Rosa Loffredo, Bruno Casciaro, Loretta Ferrera, Floriana Cappiello

**Affiliations:** 1 Laboratory Affiliated to Pasteur Italia-Fondazione Cenci Bolognetti, Department of Biochemical Sciences, Sapienza University of Rome, 00185 Rome, Italy; mariarosa.loffredo@uniroma1.it (M.R.L.); bruno.casciaro@uniroma1.it (B.C.); floriana.cappiello@uniroma1.it (F.C.); 2 UOC Genetica Medica, IRCCS Istituto Giannina Gaslini, 16147 Genoa, Italy; lorettaferrera@gaslini.org

**Keywords:** antimicrobial peptides, frog skin, D-amino acids, *Pseudomonas aeruginosa* infections, cystic fibrosis, wound healing, delivery systems

## Abstract

Antimicrobial resistance is a silent pandemic harming human health, and *Pseudomonas aeruginosa* is the most common bacterium responsible for chronic pulmonary and eye infections. Antimicrobial peptides (AMPs) represent promising alternatives to conventional antibiotics. In this review, the in vitro/in vivo activities of the frog skin-derived AMP Esc(1-21) are shown. Esc(1-21) rapidly kills both the planktonic and sessile forms of *P. aeruginosa* and stimulates migration of epithelial cells, likely favoring repair of damaged tissue. However, to undertake preclinical studies, some drawbacks of AMPs (cytotoxicity, poor biostability, and limited delivery to the target site) must be overcome. For this purpose, the stereochemistry of two amino acids of Esc(1-21) was changed to obtain the diastereomer Esc(1-21)-1c, which is more stable, less cytotoxic, and more efficient in treating *P. aeruginosa*-induced lung and cornea infections in mouse models. Incorporation of these peptides (Esc peptides) into nanoparticles or immobilization to a medical device (contact lens) was revealed to be an effective strategy to ameliorate and/or to prolong the peptides’ antimicrobial efficacy. Overall, these data make Esc peptides encouraging candidates for novel multifunctional drugs to treat lung pathology especially in patients with cystic fibrosis and eye dysfunctions, characterized by both tissue injury and bacterial infection.

## 1. Introduction

Antimicrobial resistance is one of the major threats to global human health and is predicted to be the next pandemic, because of the growing incidence of microorganisms (e.g., the Gram-positive bacteria belonging to *Staphylococcus* and *Streptococcus* spp., as well as the Gram-negative bacterium *Pseudomonas aeruginosa* [1,2,3]) that have become unresponsive to traditional antibiotics [4,5]. The alarming danger of antibiotic resistance had already been foreseen by the first discover of antibiotics, Alexander Fleming. Indeed, in 1945, during his Nobel Prize acceptance speech, Fleming admitted that if “*penicillin needs to be used, use the highest possible dosage*”, since bacteria have to be killed; “*otherwise, antimicrobial resistance is developed*” [6]. According to the last World Health Organization reports, the number of annual deaths due to drug-resistant infections (around 700,000), is expected to grow up to tenfold by 2050, leading to approximately 10 million deaths per year worldwide, compared to the five million caused by the COVID-19 pandemic [7]. This means that one person would die every three seconds if no action against antimicrobial resistance is taken, if new antimicrobial compounds are not discovered, or if their usage is not correct. In other words, we could return to the pre-antibiotic era with any suitable weapons to defeat multidrug-resistant bacterial species, also defined as superbugs or ESKAPEE pathogens [8,9]. Among these latter, *P. aeruginosa* is a highly virulent bacterium that is very difficult to eradicate [10,11,12]. In fact, aside from possessing a high level of intrinsic resistance to most antibiotics, it also has the ability to adhere to biological and inert surfaces, such as catheters or contact lenses (CLs), forming sessile communities named biofilms [13,14,15]. In biofilms, bacterial cells are embedded into an extracellular matrix produced by themselves and enter into a dormant, metabolically-inactive state, which confers protection from traditional antibiotics that usually act on the biological processes of bacterial cells controlling nucleic acids, proteins, or cell wall synthesis [16,17,18].

## 2. *Pseudomonas aeruginosa* Infections: Pneumonia and Keratitis

*Pseudomonas* biofilm formation is responsible for a large variety of severe infections, like those found in the lungs of cystic fibrosis (CF) patients or associated with the ocular surface, like keratitis, especially in CL wearers [19,20]. CF is a genetic disorder characterized by mutations in the gene encoding the CF Transmembrane conductance Regulator (CFTR), which controls the passage of chloride ions through the membrane of epithelial cells, including the airway epithelium [20,21]. The most common mutation causes the loss of phenylalanine 508 (F508del-CFTR) and the production of a misfolded protein that is rapidly degraded [22]. Because of this trafficking defect, only a small fraction of the mutated protein reaches the apical membrane of epithelial cells [23]. In addition, the mutated F508del channel has a gating defect [24,25]. As a result, the extracellular flux of chloride ions is inhibited, followed by increasing water absorption by epithelial cells with the formation of a sticky and dehydrated mucus coating the airways [26]. This favors the entrapment and accumulation of inhaled microbes, e.g., *P. aeruginosa*, which start colonizing the lung environment to establish a chronic infection accompanied by lung tissue damage and the final failure of respiratory functions [26]. Based on that, an ideal treatment of lung pathology in CF may benefit from a compound endowed with multiple biological functions, not only antimicrobial. In comparison, bacterial keratitis is the second cause of blindness after cataracts [27]. It is provoked by infection of the cornea, and *P. aeruginosa* is the most common microbial pathogen to settle on an injured ocular surface [28]. Therefore, the discovery of novel drugs to prevent and treat these types of infections has become highly demanded; and frog skin-derived antimicrobial peptides (AMPs) are promising alternatives to antibiotics [29,30,31], as reported in the paragraphs below.

## 3. Antimicrobial Peptides (AMPs)

AMPs are gene-encoded molecules containing from 10 to 50 amino acids, generally produced as prepropeptides [32,33] by almost all living organisms as key components of the host innate immune system [34]. Although AMPs differ in their chain length, sequence, and secondary structure, most of them are cationic molecules at physiological pH, with an amphipathic character in a membrane-mimicking environment, which are two fundamental features for their mechanism of microbial killing [35,36]. Indeed, especially for the cationic alpha-helical AMPs, this mechanism is based on an initial electrostatic interaction with the negatively-charged components of the microbial cell surface (like lipopolysaccharides, LPS, in Gram-negative bacteria), to subsequently reach the target bacterial cytoplasmic membrane that is much richer in anionic phospholipids, i.e., phosphatidylglycerol and cardiolipin, than the membrane of mammalian cells, which is mainly made of electrically neutral counterparts [37]. This difference is one of the main reasons accounting for the preferential activity of AMPs towards microbial cells [38]. Afterward, the bacterial membrane is permeabilized by pore formation or disintegrated in a carpet-like manner leading to cell death [39], a highly disruptive event that restricts the induction of microbial resistance. In contrast, traditional antibiotics usually interfere with biochemical reactions upon interaction with a single stereospecific target [40]. In most cases, this latter is an enzyme, and its mutations make it easier for the microbes to became resistant to conventional antibiotics that would no longer be able to recognize their specific and single target once modified [41].

Notably, apart from displaying antimicrobial activity by direct killing of microbes, some AMPs do have immunomodulatory properties that indirectly contribute to the pathogens’ clearance of the host by recruiting inflammatory cells to the site of infection, by stimulating immune cell differentiation, LPS neutralization [42], suppression of proinflammatory cytokine release [43], and promotion of wound healing [44,45,46]. This is the reason for referring to these molecules as host defense peptides [33], and the models that have been proposed to explain the effect of AMPs on mammalian cells mainly rely on their direct or indirect interaction with membrane receptors of host cells [32].

**Figure 1:**
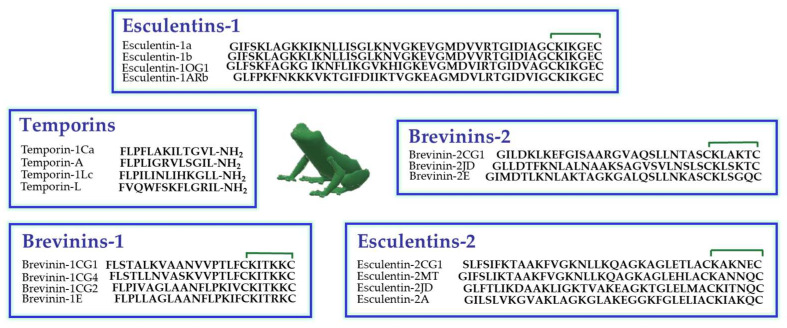
Primary structure of some members of the principal families of frog skin AMPs belonging to the Ranidae family. The presence of a disulfide bridge between the two C-terminal cysteine residues is indicated by the green line.

Amphibian skin is the most abundant natural source of AMPs, especially the skin of the Ranidae family [47]. In these animals, AMPs are synthesized and stored in dermal granular glands from which they are secreted on the skin surface upon skin lesions or stressful conditions [48]. In Figure 1, the primary structure of some members of the principal classes of frog skin AMPs, are reported. They encompass temporins (13 residues long) [49], among the shortest AMPs found in nature to date, and esculentins (up to 46 amino acids), among the longest ones [50].

## 4. Esc(1-21): Antimicrobial Activity

**Figure 2:**
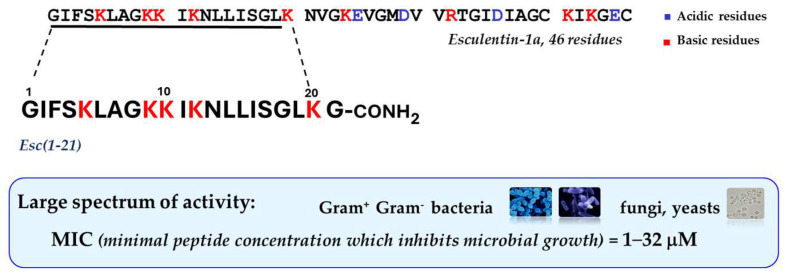
Primary structure of Esc(1-21) and its main functional properties.

In recent years, special attention has been devoted to the frog skin-derived AMP Esc(1-21). As shown in Figure 2 it corresponds to the N-terminal portion of the longer peptide esculentin-1a from the skin of the green frog living in Italy, *Phelophylax lessonae/ridibundus*, plus a glycinamide at its C-terminus [51]; it has a net charge of +6 at neutral pH, and it adopts an α-helical structure in lipid vesicles, simulating the anionic bacterial membrane [52]. 

This peptide is mainly active against Gram-negative bacteria especially *P. aeruginosa*, with minimum inhibitory concentrations (MIC) ranging from 2 to 8 μM, comparable to those of the clinically-used colistin, as reference [53]. Note that this concentration range showing antimicrobial activity in vitro overlaps the estimated concentration of peptides in the skin secretion of frogs [54], in contrast with most mammalian AMPs that display in vitro microbicidal activity at much higher concentrations than those present in physiological fluids [55]. This suggests that mammalian AMPs do function as immunomodulators rather than as weapons against microbial infections. Unlike traditional antibiotics that are not active against biofilms and that slowly kill microbes, Esc(1-21) has a fast rate of killing with a concomitant membrane-perturbation process, as proved by the extracellular leakage of a bulky cytosolic compound, such as β-galactosidase (Stokes radius of 7 nm [56]), from *Pseudomonas* cells, 15 min after peptide addition [51]. This is concomitant with a gradual reduction in the amount of viable cells, showing that the extent of membrane damage induced by the peptide increases in parallel with its concentration [51]. Scanning electron microscopy images highlighted a remarkable modification in the shape of these cells having blebs and debris from them, immediately after peptide treatment compared to control samples [51]. Contrary to the behavior described for most AMPs, similar results were obtained on the sessile phenotype of *P. aeruginosa* (even if the membrane destabilization was weaker), with disassembly of the extracellular matrix, 2 h after peptide treatment [51]. 

## 5. Esc(1-21): Non-Direct Antimicrobial Functions

**Figure 3:**
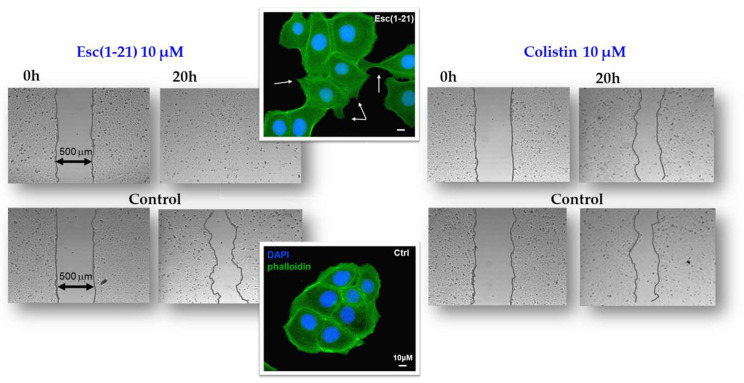
In vitro cell migration assay in bronchial epithelial cells. Esc(1-21) and colistin were evaluated for their ability to stimulate the closure of a pseudo-wound field produced in a monolayer of human bronchial epithelial cells. Peptide-treated cells, fixed in formaldehyde and stained with 4′,6-diamidino-2-phenylindole (DAPI) and phalloidin, showed cytoplasmic protrusions, indicated by the white arrows. Scale bars, 10 μm.

**Figure 4:**
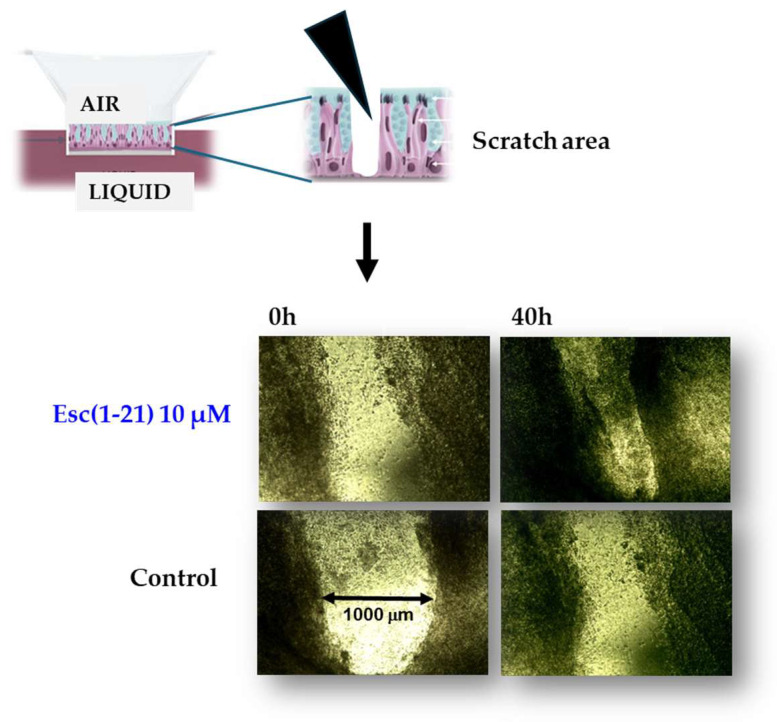
Pseudo wound healing activity of Esc(1-21) in the bronchial epithelium (primary cells from a CF patient homozygous for F508del-CFTR).

Among other activities that indirectly concur with the elimination of invading microorganisms, Esc(1-21) is able to stimulate migration of human bronchial epithelial cells, promoting the closure of a gap produced in a monolayer of these cells, within 20 h, at the optimal concentration of 10 μM [57]. This was corroborated by fluorescence microscopy studies emphasizing an altered organization of actin filaments and the presence of cytosolic protrusions, which are the typical morphological changes of cell motility, with respect to rounded untreated cells [57]. At variance, no effect on cell migration was detected for colistin, used for comparison (Figure 3). Furthermore, the wound healing activity of Esc(1-21) was preserved in the bronchial epithelium derived from a lung explant of CF patients homozygous for F508del mutation. As depicted in Figure 4, the scratch area created in this epithelium grown at the air–liquid interface became significantly smaller after exposure to the peptide, compared to the untreated epithelium. All together these findings show that Esc(1-21) is also able to accelerate the healing of injuries produced at the level of lung tissue, mostly upon persistent infections, especially in CF patients where the airway wound repair is highly compromised [58]. Concerning the subtending molecular mechanism, previous studies indicated that the wound healing process is mediated by activation of the epidermal growth factor membrane receptor (EGFR). This would trigger the intracellular signaling pathway ending with an increased production of both interleukin-8, IL-8 (playing a role in the wound healing processes [59]), and the metalloprotease MMP-9 [60,61]. In turn, MMP-9 may contribute to the disruption of the extracellular matrix as well as to the transactivation of EGFR, by catalyzing the cleavage of proligands of EGFR, as already demonstrated for the wound healing activity of the human AMP LL-37 on human keratinocytes [60]. 

The capability of Esc(1-21) to rapidly kill the planktonic and sessile forms of *P. aeruginosa*, together with the capability to promote tissue healing by eliciting cell migration, do not belong to traditional antibiotics; however, they are extremely advantageous features for the development of new anti-infective agents. In fact, an efficacious treatment of a damaged infected tissue implies not only the removal of invading infectious microorganisms but also the recovery of tissue integrity, which is expected to prevent pathogen penetration. 

## 6. AMPs’ Drawbacks for Preclinical Studies: The Design and Biological Properties of Esc(1-21)-1c

**Figure 5:**
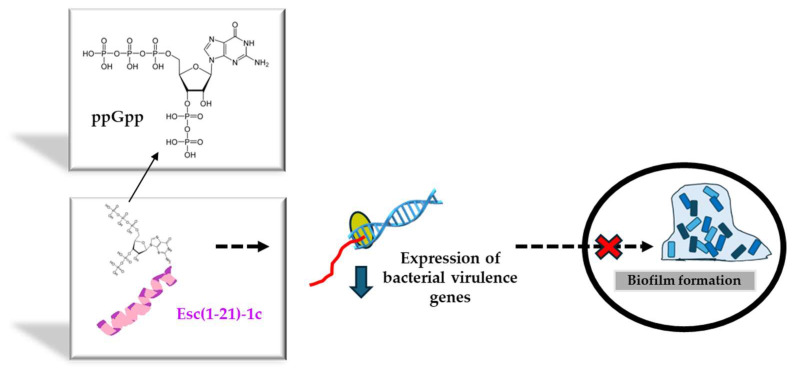
Schematic representation of the inhibition of biofilm formation by Esc(1-21)-1c upon interaction with the alarmone ppGpp. The sequester of ppGpp would reduce the expression of genes controlling biofilm formation, thus interfering with the formation of a sessile bacterial community.

Before bringing AMPs from the bench to the bedside, several drawbacks need to be overcome. Among them (i) the peptide’s cytotoxicity, (ii) the poor biostability, (iii) the limited diffusion to the target site, and the (iv) safety profile of the peptide at the site of action, especially when used at concentrations higher than the therapeutic dosages. Regarding the first two problems, a very simple strategy to circumvent them is given by the introduction of D-amino acids in the peptide sequence, which are well-known α-helical breakers, resistant to animal proteases [62]. According to the literature, there is a direct correlation between the alpha-helical structure of a peptide and its membrane-perturbing activity and cytotoxicity [52]. This latter can be reduced by lowering the peptide’s helical content upon incorporation of D-amino acids at a proper distance to break at least one turn of the alpha-helical structure [63]. Hence, in recent years, the stereochemistry of two amino acids of Esc(1-21) was changed, by replacing L-Leu^14^ and L-Ser^17^ with the corresponding D-enantiomers, to obtain the diastereomer Esc(1-21)-1c. Both peptide isoforms were designated as Esc peptides. As pointed out by NMR studies carried out on dodecylphosphocholine micelles to simulate the neutral membrane of the cells, this modification disrupted the alpha-helical structure of the parental peptide downstream asparagine residue at position 13 [52]. It also caused a drastic decrease in the noxious effect of the peptide on different types of mammalian cells, displaying a cytotoxic concentration (CC_50_) much higher than that of the all-L peptide (256 μM versus 64 μM for the all-L isoform) [64]. Furthermore, the two amino acids’ substitution was sufficient to significantly increase the peptide resistance to proteases that are abundant in the lung of CF patients, e.g., elastase from human neutrophils or from *P. aeruginosa*. While the all-L peptide was completely degraded 5 h after incubation with the two enzymes, more than 70% of the whole peptide was recorded in the case of Esc(1-21)-1c [57]. In addition, with reference to the biological activity, it was demonstrated that the presence of the two D-amino acids was sufficient to make the peptide more active against *Pseudomonas* biofilm, causing 95% biofilm eradication at lower concentrations than those needed for the all-L isomer [64]. This is likely due to the higher resistance of the diastereomer Esc(1-21)-1c to bacterial proteases that are mainly secreted by biofilm cells rather than planktonic ones [65]. In comparison to the all-L isoform, the diastereomer Esc(1-21)-1c was also found to inhibit the biofilm formation of *Pseudomonas* when used at sub-inhibitory concentrations that do not affect bacterial viability [66]. To explain this event, gene expression analysis provided the first evidence of the capability of Esc(1-21)-1c to induce a substantial reduction in the expression level of a large variety of virulence genes, encompassing the gene encoding the chemical mediator of Las I quorum sensing [66], the gene encoding Las B protease, responsible for the host tissue damage, as well as the genes implicated in the biogenesis of flagella and pili. Flagella and pili are the two major bacterial appendices that account for bacterial swimming motility and adhesion to surfaces, respectively [66]. A plausible hypothesis is that Esc(1-21)-1c hampers the biofilm formation of *Pseudomonas* by interfering with the bacterial swimming motility and with the bacterial capacity to colonize a surface and to settle a sessile community. This was corroborated by the smaller swimming zone of *Pseudomonas* in appropriate agarose plates supplemented with Esc(1-21)-1c, versus the results obtained with the all-L peptide or the control sample [66]. By employing the rhodamine-labeled Esc(1-21)-1c and confocal microscopy to visualize the distribution of the peptide (at sub-MIC) within *Pseudomonas* cells, Esc(1-21)-1c mainly accumulated at the membrane level; however, it also diffused inside the cytosol within the first 5 min [66]. In comparison, no fluorescence intensity was detected in cells treated with rhodamine alone, ruling out that the fluorophore by itself would help the translocation of the peptide into the bacterial cytosol to affect the gene expression profile, likely upon interaction with the alarmone guanosine tetraphosphate (ppGpp) to which Esc(1-21)-1c was proved to bind [66]. The alarmone ppGpp is generally produced by *Pseudomonas* cells under stressful conditions, for example, in nutrient-limited media, and it controls bacterial virulence by activation of quorum-sensing systems [67]. The binding of the peptide to ppGpp presumably leads to a reduced availability of free nucleotide molecules, thus lowering the expression level of virulence genes including the genes involved in bacterial motility, with the final inhibition of biofilm formation (Figure 5). However, we cannot exclude the participation and contribution of other signaling pathways.

In a recent paper, Esc(1-21)-1c at its sub-MIC was reported to potentiate the ability of a large variety of antibiotics to prevent the growth of *P. aeruginosa*, as demonstrated by the corresponding fractional inhibitory concentration (FIC) indexes, lower than 0.5, meaning a synergistic effect [68]. Through differential proteomic analysis and gene expression studies, researchers discovered the capability of sub-MIC doses of Esc(1-21)-1c to drop the production of mexA/B/oprM efflux pump, at both mRNA and protein levels. This outcome would limit the extrusion of antibiotics through the efflux pump and would increase their intracellular amount, as confirmed by direct measurements of antibiotics, i.e., tetracycline, within *Pseudomonas* cells after exposure to sub-MIC of Esc(1-21)-1c. Importantly, this is expected to make bacteria more susceptible to antibiotics, opening the avenues to re-evaluate the usage of old drugs, almost abandoned, in clinical practice.

## 7. Esc Peptides and CFTR Potentiator Activity in the Bronchial Epithelium

**Figure 6:**
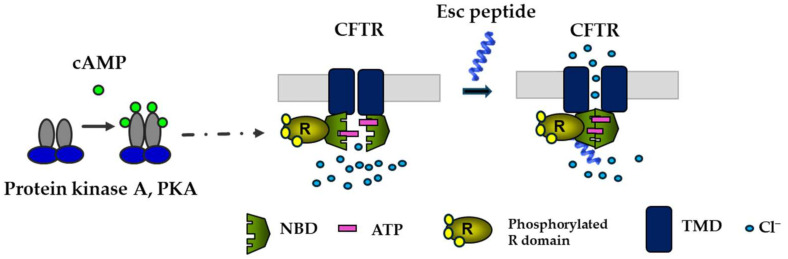
Schematic representation of the activation of CFTR with gating mutations by Esc peptides. By binding to the phosphorylated CFTR (the phosphorylation of the R domain is mediated by cAMP-dependent PKA), the peptide would provoke the dimerization of the ATP-bound NBD domains with the opening of the channel at the transmembrane domains (TMD).

Given the importance of CFTR and the airway epithelium in preserving pulmonary respiratory function and in repairing injured lung tissue [69], it was natural to investigate the effects of Esc peptides on the ion currents regulated by CFTR. To be functional, CFTR requires phosphorylation at multiple sites in the R domain, by cyclic AMP-dependent protein kinase A (PKA), as well as the binding of two ATP molecules at the nucleotide binding domains (NBDs) to facilitate their dimerization with subsequent opening of the channel at the transmembrane domains (Figure 6) [70,71]. In laboratory settings, complete activation of CFTR with gating mutations can be achieved by adding two compounds: forskolin (FSK), which raises intracellular cAMP levels promoting channel phosphorylation, and genistein (GEN), an isoflavone that enhances the probability of the channel being open, by stabilizing NBD dimerization [70].

Epithelial cells were preincubated with lumacaftor, a corrector that acts as a protein-folding chaperone to assist the delivery of the mutated protein to the cell membrane. They were then treated with a combination of FSK plus Esc peptides at different concentrations or with GEN as a positive control. As previously reported [24], a significant rise in conductance was obtained when the two peptides were used at increasing concentrations in the presence of FSK, compared to the values obtained for samples treated only with FSK. This increase was particularly pronounced for Esc(1-21)-1c at 10 μM, similar to the effect of GEN, suggesting that Esc peptides have a CFTR potentiator effect on the mutated channel (Figure 6). To understand the mechanism behind this activation, patch–clamp experiments were conducted on Fischer rat thyroid (FRT) cells using the inside-out configuration, which is a cell-free system, lacking many cellular components [24]. These experiments showed a significant enhancement in the ion current upon peptide administration, while the addition of PKA plus ATP was not enough to activate the mutated channel without the presence of any peptide [24]. Moreover, the ion current measured upon CFTR inhibition reverted to the initial value, indicating that the raised ion flux induced by the peptides is due to the activation of CFTR, likely through the direct interaction of the peptides with the channel itself (Figure 6) rather than with other cellular components, which are not expected to be present in the inside-out patch clamp configuration [24]. Remarkably, this is an unprecedented property of AMPs and contributes to making them even more attractive compounds for the development of new multifunctional therapeutic agents to treat lung pathology in CF.

## 8. In Vivo Efficacy of Esc(1-21)-1c against *P. aeruginosa* Lung Infection

**Figure 7:**
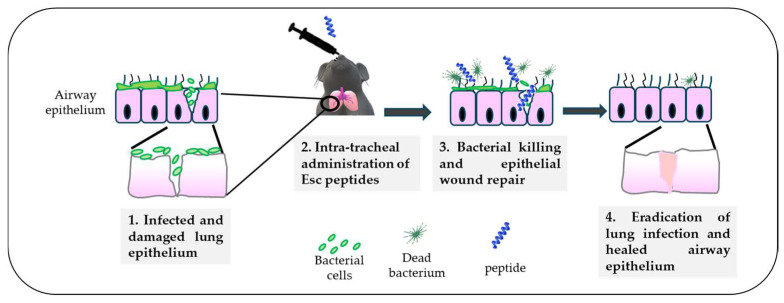
Schematic representation of the in vivo antimicrobial efficacy of Esc(1-21)-1c upon intratracheal administration in a mouse model of acute lung infection and the expected repair of the airway epithelium.

Altogether, the higher biostability of Esc(1-21)-1c jointly with its lower cytotoxicity and better biological/antibiofilm activities contributed to consideration of this peptide as the most suitable molecule for the treatment of lung infection. Nevertheless, before moving to human beings, experiments with animals, e.g., mammals like mice, provide a valid support. By means of a mouse model of acute *Pseudomonas* lung infection, it was demonstrated that a single intratracheal (i.t.) instillation of the diastereomer Esc(1-21)-1c at the low dosage of 20 μM (0.1 mg/kg), was able to produce a 2 log_10_ reduction in the lung bacterial burden, 24 h after infection, as recorded for colistin [53]. However, differently from Esc(1-21)-1c, which is also expected to promote in vivo lung epithelium wound repair (Figure 7), colistin is active only against Gram-negative bacteria, it induces resistance [72], and it does not have any airway epithelium wound healing activity to restore tissue entirety and respiratory functions, impeding pathogen penetration. 

## 9. Esc Peptides and Pulmonary Delivery Systems

**Figure 8:**
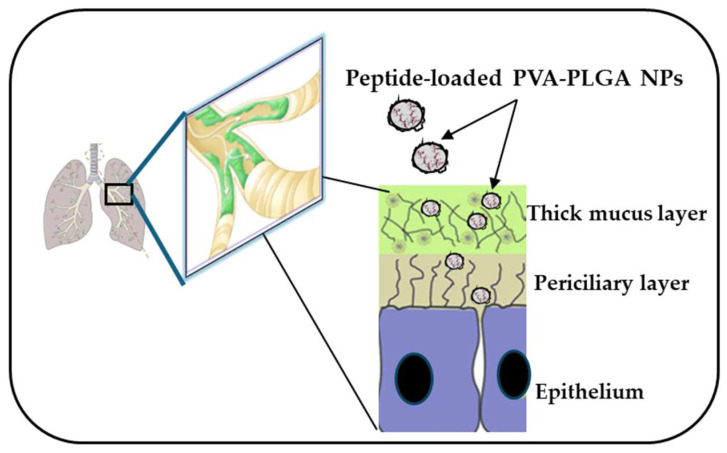
PVA-PLGA NPs loaded with Esc peptides have the ability to diffuse through biological barriers, such as the lung mucus imposed by lungs that becomes a thick layer in CF patients.

As mentioned above, aside from cytotoxicity and biostability, another issue that needs to be circumvented for the clinical translation of AMPs is their limited diffusion through biological barriers and limited delivery to the target site, such as the lungs. Due to advances in nanotechnologies, biodegradable polymeric nanoparticles (NPs) made of poly(lactide-coglycolide) (PLGA), loaded with each Esc peptide at 2% (*w*:*w*), coated with the hydrophilic polymer polyvinyl alcohol (PVA), were revealed to be a suitable nanocarrier to assist the diffusion of the encapsulated AMP through those bio-barriers imposed by lungs, such as bronchial mucus (Figure 8) [73,74]. These NPs were prepared according to the solvent/diffusion technique and had a hydrodynamic diameter lower than 300 nm, a suitable size to reach the deepest part of the lungs, i.e., the alveoli [75,76]. Furthermore, these NPs were found to prolong the antibacterial activity of the encapsulated peptide. In line with that, although the free Esc(1-21) completely inhibited bacterial growth within a short time (24 h), this effect was lost over a longer term, for example, after 72 h. In comparison, a constant, even if weaker antimicrobial activity was manifested by the peptide-loaded NPs, supposedly due to the gradual release of the peptide from the NPs. This would prolong the peptide residence time, maintaining a constant concentration of the peptide at the site of action over time (some peptide molecules would be degraded, some others would be released from the NPs), thus extending the peptide’s antimicrobial efficacy [77]. In addition, these NPs were able to potentiate the in vivo antimicrobial efficacy of the encapsulated Esc peptides, in the lung, upon administration in the conductive airways, causing a more marked reduction in the lung bacterial burden compared to the results obtained for the corresponding peptides, when administered in their soluble free form.

## 10. Esc Peptides and Pulmonary Safety Profile

The fourth problem that needs to be solved before proposing AMPs or AMP-based NPs as future drugs for therapeutic purposes in humans is their safety profile in terms of gene expression and tissue integrity at the target site, especially when used at concentrations higher than the therapeutic dosages. This aspect has not been explored for most AMPs. Interestingly, differential gene expression analysis from RNA sequencing did not show any global genetic change in the lung tissue of healthy mice, 24 h after i.t. administration of Esc peptides of 0.1 mg/kg in the free or encapsulated form (no more than six up- or downregulated genes compared to the vehicle-treated animals) [78]. Yet, the most efficient, Esc(1-21)-1c, did not elicit any pulmonary proinflammatory effect or damage either after 1 day or 14 days from its i.t. instillation in the free or encapsulated form at a concentration 15-fold higher the therapeutic concentration [78]. In parallel, no toxicity from the bare NPs was appreciated, thus excluding that the NPs can affect the protective functions of the lung [78]. Finally, the most efficient isoform Esc(1-21)-1c was well tolerated by animals, who remained alive with the same movement ability as the untreated control mice, either after 1 h or 24 h from its i.t. administration at a concentration 70-fold higher than the efficacy dose, without provoking any visible ruptures in the lungs or in other organs, like the spleen, kidney, and liver. Furthermore, this peptide is not immunogenic in mice [61], strengthening its safety profile for clinical translation.

**Figure 9:**
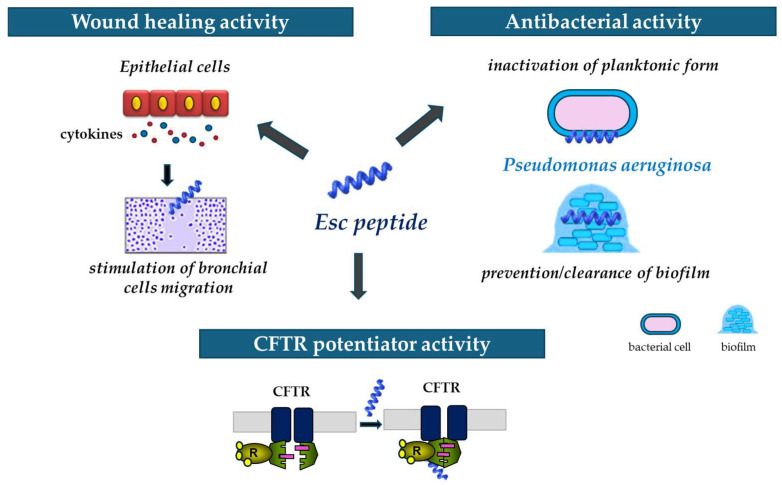
Schematic representation of the multiple properties of Esc peptides: (i) ability to stimulate migration of epithelial cells, thus favoring the wound healing activity; (ii) antibacterial activity against the planktonic and biofilm forms of *P. aeruginosa;* and (iii) ability to potentiate the activity and opening of the mutated CFTR.

In summary, it can be stated that Esc peptides, particularly Esc(1-21)-1c do represent encouraging candidates for new strategies to treat *Pseudomonas* lung infection especially in CF patients, able to act not only as antibiotic agents but also as wound healing promoters and potentiators of CFTR with conductance defects (Figure 9).

## 11. Esc Peptides’ Efficacy towards *P. aeruginosa* Keratitis

**Figure 10:**
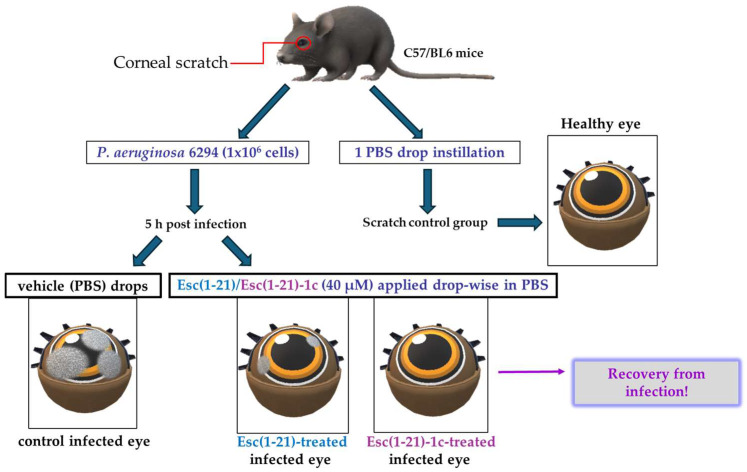
Schematic representation of the experimental procedure to evaluate the in vivo antipseudomonal activity in a mouse model of *P. aeruginosa*-induced keratitis.

Keratitis is an infection of the cornea, the transparent membrane covering the eye that contributes not only to refraction of the light but also to the ocular surface immune response [79]. Keratitis generally occurs upon corneal abrasion, due to accidental trauma or CL wear [27,80,81,82]. One of the major threats for the usage of AMPs to treat eye infections is their inactivation by tear film components [83]. The tear film continually bathes the ocular surface providing lubrification, protection, and nutrients to the cornea; it contains (i) a lipid layer in contact with the air that stops tears from evaporating, (ii) an aqueous layer rich in salts, (iii) and a mucus layer adjacent to the cornea. Initially, the peptide Esc(1-21) was investigated for its in vitro anti-pseudomonal activity in the presence of sodium chloride as well as in the presence of basal human tears. Surprisingly, in contrast with most human AMPs [83,84], Esc(1-21) was found to fully retain its bactericidal activity against *P. aeruginosa* at salt concentrations up to 150 mM, comparable to the salt concentration in tear fluid. Most importantly, such activity was well preserved in the presence of 70% basal human tears at 1 μM, unlike the human AMP hBD2, produced by the cornea [84], which loses its activity under such conditions, as well as at the higher peptide concentration of 20 μM [85]. These exciting results prompted in vivo studies in a mouse model of keratitis. The cornea of the left eye of mice was scratched with a needle and infected with *P. aeruginosa*. Five hours after infection, both Esc peptides were locally applied drop-wise to the ocular surface at 40 μM, 3 times per day for 3 days (whereas regular antibiotics are administered every 15 min during the first days of treatment). Control animals received the vehicle (physiological solution) to the infected eyes, whereas the scratched uninfected mice only received 1 drop instillation of a physiological solution (Figure 10). The disease severity was assessed by slit-lamp examination according to a standardized grading scale from level 0 (clear transparency of the eye) to level 4 (corneal perforation). The control eyes showed a clear infection, whereas the peptide treatment significantly reduced the level of keratitis with a higher efficacy for the diastereomer Esc(1-21)-1c, allowing recovery from the infection within only two days (Figure 10). While a dense opacity fully covering the pupil and cornea was observed in the control infected eyes, only a slight opacity partially covering the ocular surface or a complete transparency similar to that found in the scratched uninfected eyes were noted two days after treatment with the all-L peptide or the diastereomer, respectively [86].

So far only a few studies have been carried out with animal models of microbial keratitis and AMPs giving signs of clinical benefits, despite their strong in vitro activity against a large variety of ocular surface microbial pathogens [87]. Lately, it was discovered that the most efficient peptide Esc(1-21)-1c can also speed up the healing process of a circular cornea wound, within 24 h, after a double instillation at the same concentration range displaying in vivo efficacy against *Pseudomonas* keratitis [86]. Promotion of re-epithelialization of an injured cornea is an essential process not only for refraction of the light but also to forestall pathogen infiltration and therefore the occurrence of ocular surface microbial infections [79,88]. These data are also in line with the results of in vitro experiments highlighting the capability of Esc(1-21)-1c to stimulate the migration of human corneal epithelial cells within 24 h and to elicit the secretion of proinflammatory cytokines, like IL-6, or growth factors that play a fundamental role in the regulation of corneal wound healing [89]. 

Unfortunately, *Pseudomonas*-induced keratitis is mainly associated with CL wear because of the propensity of this pathogen to adhere quickly to soft CLs, forming biofilm [80,90]. Once a contaminated CL is placed in the eye, the bacterium rapidly spreads through the cornea, causing an inflammation process and vision loss if the infected cornea is not treated within 2–3 days. It should be considered that, in CL wearers, there is a lower production of antimicrobial compounds at the level of the cornea and a lower production of tear fluid to mechanically remove foreign particles [91]. It has been estimated that the incidence of keratitis is about 5–20 people per 10,000 CL wearers and that about two thousand people suffer from this disease [80]. CL wear is one of the most popular ways to correct different types of vision disorders, providing better vision than glasses and improved performance in sports activities. Among the most common risk factors for microbial keratitis is the incorrect handling of this medical device, including prolonged wear of disposable CLs, poor hygiene conditions, CL wearing during swimming, or the usage of cosmetic CLs.

**Figure 11:**
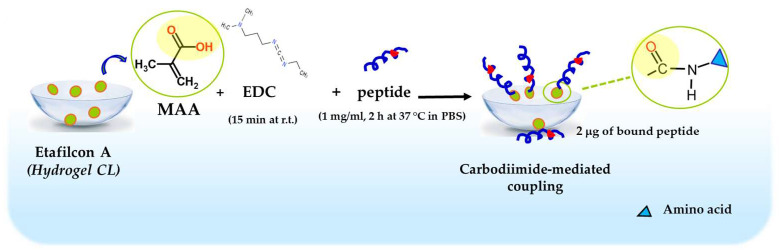
Schematic representation of Esc peptides’ conjugation to hydrogel soft CLs. MAA, methacrylic acid; EDC, 1-Ethyl-3-(3dimethylaminopropyl)carbodiimide; PBS, phosphate buffered saline; r.t., room temperature.

Taking advantage of the information in the literature for melamine-coated lenses [92], Esc peptides were immobilized to hydrogel soft CLs (Etafilcon A, containing methacrylic acid, MAA). The carboxylic group of MAA was activated with 1-ethyl-3-(3-dimethylaminopropyl) carbodiimide hydrochloride (EDC) and subsequently functionalized with the peptides through a carbodiimide-mediated coupling to form an amide bond (Figure 11).

**Figure 12:**
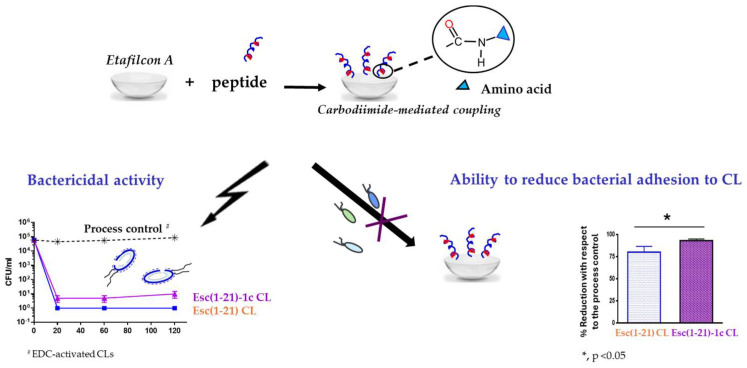
Advantageous properties of Esc-peptides coated CLs: bactericidal activity against the planktonic form of *P. aeruginosa* ATCC 27853, as indicated by the number of colony forming unit (CFU)/mL (**left side**) and effect on bacterial adhesion on the CL surface (**right side**) in comparison to the process control, EDC-activated CLs.

Using a peptide solution of 1 mg/mL, 2 μg of bound peptide per lens was obtained. The covalent attachment of Esc peptides to soft CLs was revealed to be an effective strategy to achieve an antimicrobial surface endowed with bactericidal activity and the ability to significantly reduce bacterial adhesion to the lens surface [93]. Indeed, a higher bactericidal activity (more than 99.99% killing of bacterial cells) was displayed by CLs coated with Esc(1-21)-1c after 20 min incubation with *Pseudomonas* cells, compared to the results obtained with the corresponding peptide when used in its free form; in addition, about a 97% decrease in *P. aeruginosa* cells on Esc(1-21)-1c-coated CLs was recorded 24 h after lens incubation with the bacterial culture, compared to the EDC-treated CL (Figure 12). This is an important reduction in the bacterial transfer from the lens to the ocular surface, once the antimicrobial lens is placed on the eye, stopping the establishment of infection. Remarkably, peptide immobilization to these CLs did not make them toxic to mammalian cells and did not alter their surface parameters such as the diameter, center thickness, and curvature (which are crucial factors to keep the optical properties of the lenses) [93].

To the best of our knowledge, this is the first case showing the ability of a frog skin AMP (i) to exhibit activity in a mouse model of keratitis and to promote cornea healing in a mouse model of corneal debridement wound at the same micromolar concentration; (ii) to preserve antimicrobial activity upon immobilization to a medical device surface. As future work, it will be interesting (i) to evolve Esc peptides as new ophthalmic agents for CL storage solutions, (ii) to optimize their coating to lenses, for preventative usage and to avoid ocular surface infections, as well as (iii) to identify the best formulation to apply these peptides to the ocular surface for the treatment of eye dysfunctions, like keratitis, characterized by both tissue injury and cornea infection, thus fostering research studies in the field of peptide science for clinical trials [94,95,96].

## 12. Conclusions

**Figure 13:**
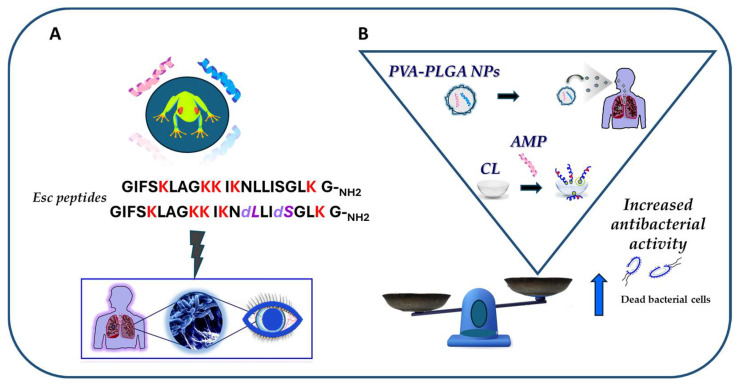
Schematic representation of the main outcomes of the review article. (**A**) The frog skin-derived Esc peptides display in vivo activity against *P. aeruginosa*-induced lung and ocular surface infections; (**B**) efficacy of nanoparticulate systems (PVA-PLGA-loaded Esc peptides and Esc peptide-coated CL) in increasing the antibacterial activity of the peptides.

In conclusion, this review has sharpened how the frog skin-derived Esc peptides, despite being short and linear molecules, represent appealing templates for the generation of new drugs against pulmonary and ocular surface infections, induced by *P. aeruginosa* and likely other microbial pathogens (Figure 13A). In comparison to the mammalian counterparts, such as the AMP LL-37, Esc peptides have the following advantages: (i) they display antibacterial activity at the same concentrations found in their natural environment; (ii) they retain antibacterial activity in biological fluids; and most importantly, (iii) they ameliorate the function of defective CFTR, which are all profitable features for novel multifunctional weapons to tackle the antimicrobial resistance pandemic. In addition, it has been recalled how nanotechnology strategies based on the incorporation of Esc peptides into engineered nanoparticulate systems (i.e., PLGA NPs) or the conjugation of Esc peptides to biomaterials (like CLs) represent an exciting approach to improve delivery and to augment the antibacterial activity of AMPs at the target site (Figure 13B). 

## Data Availability

Data are contained within the article.
